# Fabrication of Thermal Conductivity Detector Based on MEMS for Monitoring Dissolved Gases in Power Transformer

**DOI:** 10.3390/s20010106

**Published:** 2019-12-23

**Authors:** Tingliang Tan, Jianhai Sun, Tingting Chen, Xinxiao Zhang, Xiaofeng Zhu

**Affiliations:** 1 State Key Laboratory of Transducer Technology, Aerospace Information Research Institute, Chinese Academy of Sciences, Beijing 100094, China; tantingliang17@mails.ucas.ac.cn (T.T.); chentingting19@mails.ucas.ac.cn (T.C.); zhangxinxiao19@mails.ucas.ac.cn (X.Z.); 2 School of Electronic, Electrical, and Communication Engineering, University of Chinese Academy of Sciences (UCAS), Beijing 100049, China; 3 Beijing Municipal Institute of Labour Protection, Beijing 100054, China

**Keywords:** microelectromechanical system, micro-thermal conductivity detector, dissolved gases, high sensitivity

## Abstract

In this work, a high sensitivity micro-thermal conductivity detector (μTCD) with four thermal conductivity cells was proposed. Compared with conventional TCD sensors, the thermal conductivity cell in this work was designed as a streamlined structure; the thermistors were supported by a strong cantilever beam and suspended in the center of the thermal conductivity cell, which was able to greatly reduce the dead volume of the thermal conductivity cell and the heat loss of the substrate, improving the detection sensitivity. The experimental results demonstrated that the μTCD shows good stability and high sensitivity, which could rapidly detect light gases with a detection limit of 10 ppm and a quantitative repeatability of less than 1.1%.

## 1. Introduction

The gas monitoring system of transformer oil is very important to the ensure safe operation of power transformers [1,2,3]. Existing dissolved gas detection technologies mainly include photoacoustic spectroscopy, electronic nose, and other technologies. However, photoacoustic spectroscopy requires expensive optical components and lasers, and the application of this technique in dissolved gas detection is greatly limited. Electronic nose technology is relatively much more economical, but this technology is integrated by multiple sensors and low detection sensitivity, and a short sensor life limits its popularity. A thermal conductivity detector (TCD) is a broad-spectrum detector that can respond to small-molecule gases such as CO, H_2_, C_2_H_2_, and C_2_H_6_ [4,5]. However, traditional TCD is difficult to be applied to trace gas detection [6] due to its large volume and low detection sensitivity. Due to the adoption of MEMS technology, the volume of the thermal conductivity detector is reduced from the traditional micro-upgrade to the nano upgrade. Therefore, its sensitivity is greatly improved, which is an order of magnitude higher than the traditional structure. Due to its high sensitivity, this kind of sensor has attracted great attention from researchers.

Chen researched the micro-channel flow of μTCD and analyzed the influence of channel size and gas properties on the heat transfer rate in detail [7]. Cruz reported a μTCD that was capable of detecting chemical species at several ppm concentration levels [8]. Kaanta realized the monolithic integration of the micro-thermal conductivity detector and the micro-chromatographic column, which reduced the volume of the detector [9]. Narayanan designed a four-cell μTCD, but the thermistors adopted a non-suspension structure with large thermal loss, reducing the sensitivity of the detector [10].

In order to improve the sensitivity of TCD, a high sensitivity μTCD with four-cell based on MEMS is proposed in this work. The thermal conductivity cell of the sensor is consistent with the structure of the air flow channel, presenting a streamlined structure. The whole airflow channel has no diameter variation, and the thermal conductivity cell has no obvious dead volume, thus greatly improving the sensitivity of the detector. Moreover, the beam supporting the thermistors is formed by thicker low-stress silicon nitride. The thicker beam can avoid thermal stress caused by the impact of airflow and affect the stability of the detector.

## 2. Analysis and Design

**Figure 1:**
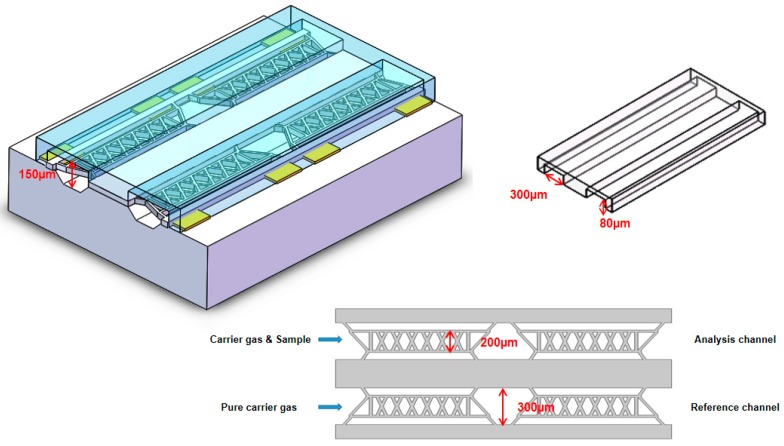
Schematic diagram of the micro-thermal conductivity detector (μTCD).

The μTCD in this paper consists of four resistors with the same resistance, and the four thermistors form a Wheatstone bridge. If only pure carrier gas is transported into the channels at the same time, the bridge is balanced because the resistance values of these thermistors are the same. When carrier gas with sample flows through the analysis channel and pure carrier gas flows through the reference channel, the balance of the bridge will be broken and an output voltage will be obtained [11]. In order to obtain higher sensitivity, four resistors supported by a beam were suspended in the center of the airflow channel (as shown in Figure 1), which was released by deep reactive ion etching (DRIE) [12] and KOH etching [8,13].

The principle of μTCD is a concentration detector that responds to the difference in thermal conductivity between the tested component and carrier gas. From the perspective of heat exchange, the heat loss of μTCD mainly includes heat conduction, heat convection, and heat radiation. Heat conduction is the main research section, while the effect of natural convection and thermal radiation on μTCD are negligible. According to the principle of heat balance, the heat generated by the current of the thermistor is equal to the total heat loss energy. The heat generated by the thermistor can be represented by the following Equation [14]:

(1) $$P=I^{2}R=I^{2}R_{0}[1+\alpha (T_{h}-T_{v})]$$

where *I* is the loading current, *R*_0_ is the resistance value of the thermistor, *T_h_* is the temperature of thermistor, *T_v_* is the temperature of fluid, and *α* is the temperature coefficient of thermistor. The heat loss also includes the heat of the thermal sensor convection to the fluid (*Q*_1_) and the heat of the thermal sensor conduction to the insulation layer (*Q*_2_). *Q*_2_ is further divided into three parts: the heat stored in the insulation layer (*Q*_2a_), the heat of convection from the insulation layer to air (*Q*_2b_), and the heat of conduction from the insulation layer to the substrate (*Q*_2c_). The *Q*_1_ can be expressed as following [15]:

(2) $$Q_{1}=hA_{s}(T_{h}-T_{v})$$

where *h* is the forced convection coefficient and *A_s_* is the contact area of gas and thermistor. The results show that the sensitivity of the sensor can be improved by increasing the proportion of *Q*_1_ [16]. Therefore, opening a cavity in the base structure of the thermal conductivity detector is helpful to increase *Q*_1_ and reduce the heat of the base or insulation layer.

## 3. Optimization and Fabrication of the μTCD

### 3.1. Configuration of μTCD

**Figure 2:**
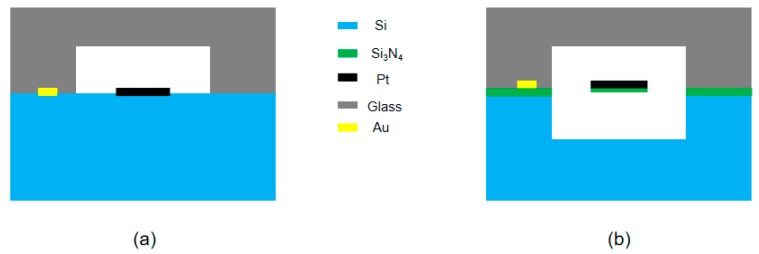
(**a**) Configuration of the conventional TCD. (**b**) Thermistors with suspended structure of μTCD.

Compared with the conventional TCD (Figure 2a) [10], the micro-thermal conductivity detector adopted a streamlined structure and the four thermistors were suspended in the center of the cell (Figure 2b), which decreases the dead volume and the heat loss of the base. The sensitivity of μTCD [17] can be expressed as:

(3) $$S=KI^{3}R\frac{\lambda_{c}-\lambda_{s}}{\lambda_{c}}(T_{f}-T_{w})$$

where *K* is the geometric constant of the thermal conductivity cell, *I* is the bridge current, *R* is the resistance, *λ_c_* is the thermal conductivity of the carrier gas, *λ_s_* is thermal conductivity of the sample gas, *T_f_* is the temperature of thermistors, and *T_w_* is the temperature in the thermal conductivity cell. According to Formula (3), the value of is *T_f_ − T_w_* is greater and the sensitivity of the detector is much higher. In this work, the sensitivity of the detector was mainly improved by reducing the temperature of the thermal conductivity cell. Therefore, the thermistors supported by a cantilever beam were suspended in the center of the cell in this design, which can reduce the heat loss of the thermistors and greatly decrease the temperature of the thermal conductivity cell.

**Figure 3:**
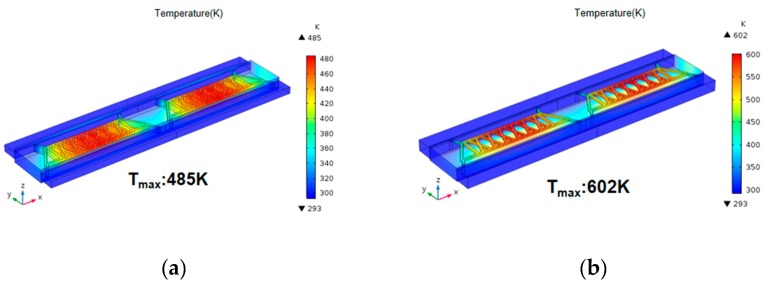
Surface temperature of thermistors: (**a**) conventional structure; (**b**) thermistors with suspended structure.

Figure 3 shows the thermal distribution of thermistors. It can be seen that the surface temperature of thermistors with suspended configuration in the cell (Figure 3a) was much higher than that of conventional structure (Figure 3b) as the heat loss of the thermistors was smaller, so the thermistors with suspended structure were able to improve the sensitivity by reducing the thermal loss of the base.

### 3.2. Optimization of Supporting Beam

**Figure 4:**
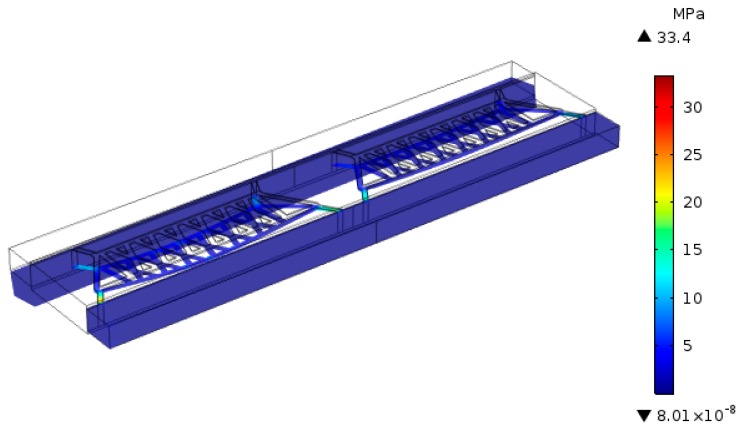
The stress on the cantilever beam under certain loading conditions.

Thermistors supported by cantilever beams were suspended in the thermal conductivity cell, which was able to reduce the heat loss of the substrate, thus improving the detection sensitivity. However, the cantilever beam will vibrate under the impact of the carrier airflow, resulting in stress and deformation, thus causing greater thermal noise. Thermal noise is an important factor affecting the sensitivity of the sensor, which will reduce the sensitivity and stability of the sensor. In order to reduce the thermal noise, the thickness of the cantilever beam is simulated. Figure 4 shows the relationship between the stress on the cantilever beam and the film thickness under certain loading conditions (constant flow rate and loading power). The results (as shown in Table 1) show that the stress decreases greatly with the increase of the thickness of the cantilever beam. Therefore, a layer of low-stress silicon nitride film in which the maximum thickness can be processed is selected as the supporting beam, improving the consistency and reliability of the detector.

### 3.3. Effects of Loading Conditions

**Figure 5:**
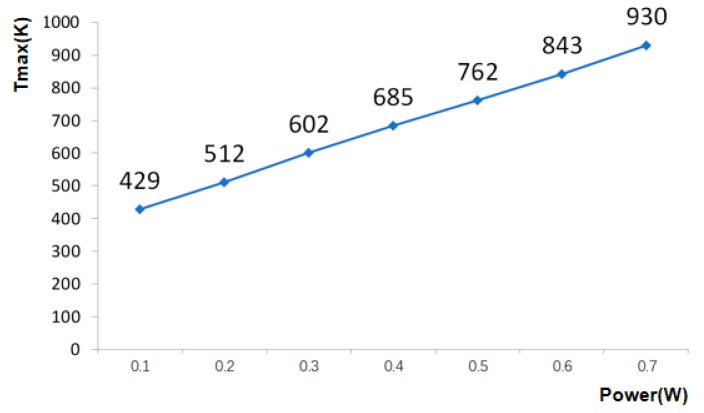
Maximum temperature of thermistors at different loading power.

According to the sensitivity of the sensor, the loading condition is a key factor. Therefore, different loading conditions were analyzed in this work. The relationship between temperature and power can be obtained as follows:

(4) $$\Delta T=\frac{Pt}{cm}$$

where *P* is power, *c* is specific heat capacity, *m* is mass, *t* is time, and *ΔT* is temperature change. Figure 5 shows the heat distribution of the thermistors with suspended structure at a constant flow rate (5 mL/min) and different power. It can be seen that the sensitivity of the detector can be rapidly improved by increasing the power. However, the life of the thermistor will be affected if the temperature is too high and, therefore, a suitable amount of power is used to drive the sensor.

In addition, the influence of carrier gas velocity on sensitivity is also evaluated. Table 2 shows the relationship between the surface stress of the thermistor and the flow rate under a constant power of the sensor. Table 3 shows the mechanical properties of the SiN [18]. The results show that the stress in the thermistor increases rapidly with the increase of the carrier gas velocity, and the increased amplitude is very large. The stress at the flow rate of 10 mL/min is five times higher than that at the flow rate of 2 mL/min. Excessive stress will cause deformation or even collapse of the supporting beam. Therefore, a small carrier gas velocity can improve the stability of the detector.

### 3.4. Fabrication of the μTCD

**Figure 6:**
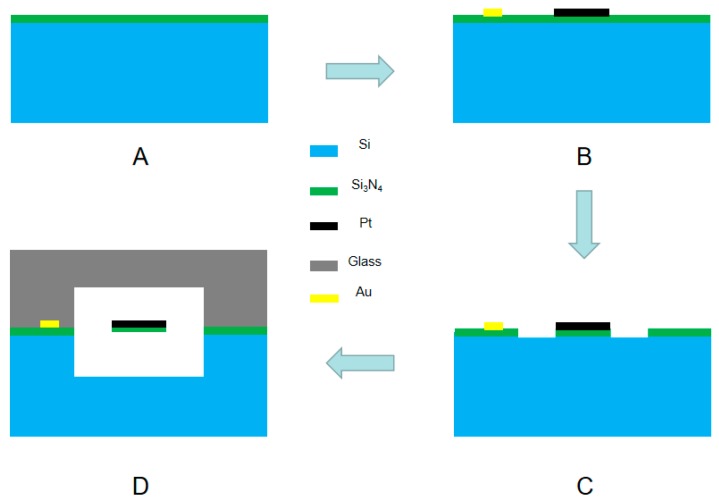
(**A**–**D**) The fabrication process of μTCD, including key steps of (**A**) deposition of SiN layer, (**B**) deposition and patterning of thermistors and electrodes, and (**C**) masking for channel etch. (**D**) After releasing supporting beam by deep reactive ion etching (DRIE), a pyrex 7740 glass is anodically bonded, completing the μTCD.

**Figure 7:**
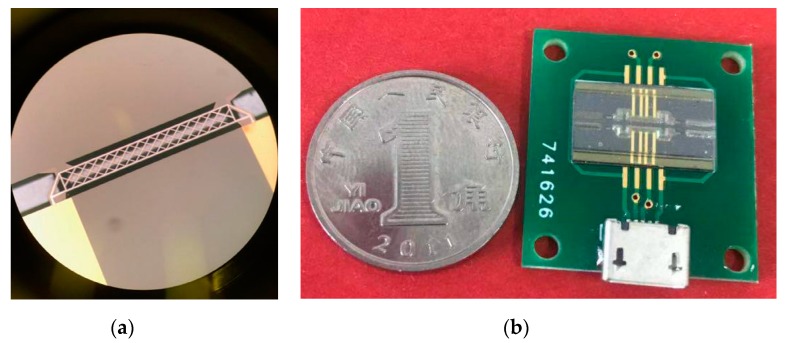
(**a**) The micrograph of μTCD. (**b**) The chip of the sensor.

The fabrication process of the sensor based on MEMS is shown in Figure 6.

(A) SiN with a thickness of 1500 nm for insulating was deposited on the surface of the silicon wafer by low-pressure chemical vapor deposition (LPCVD) after the wafer was thoroughly cleaned by boiled sulfuric acid and deionized water.

(B) At first, the mask of thermistors pattern (AZ1500 photoresist) was prepared on the surface of SiN film by photolithography and then cleaned by oxygen plasma (2 min). Cr (20 nm) and platinum Pt (200 nm) were sputtered in sequence on the patterned surface of the SiN film. Subsequently, the thermistors were formed by lift-off technology after the underneath photoresist was removed by acetone, alcohol, and deionized water. Finally, the electrodes were obtained in the same way by replacing the metal sputtered with Cr (20 nm) and Au (200 nm).

(C) For protecting the surface of thermistors and electrodes, the photoresist mask pattern (AZ4620 photoresist) was formed on the surface by photolithography. Then, SiN where there were no resistors and electrodes, were removed by trifluoromethane etching to form the supporting beam.

(D) The supporting beam was released by DRIE and potassium hydroxide etching to obtain a gas flow channel and finally bonded to the pyrex 7740 glass to obtain μTCD. Figure 7 indicates the micrograph of the μTCD and the photo of the sensor.

## 4. Results and Discussion

### 4.1. Experimental Setup

**Figure 8:**
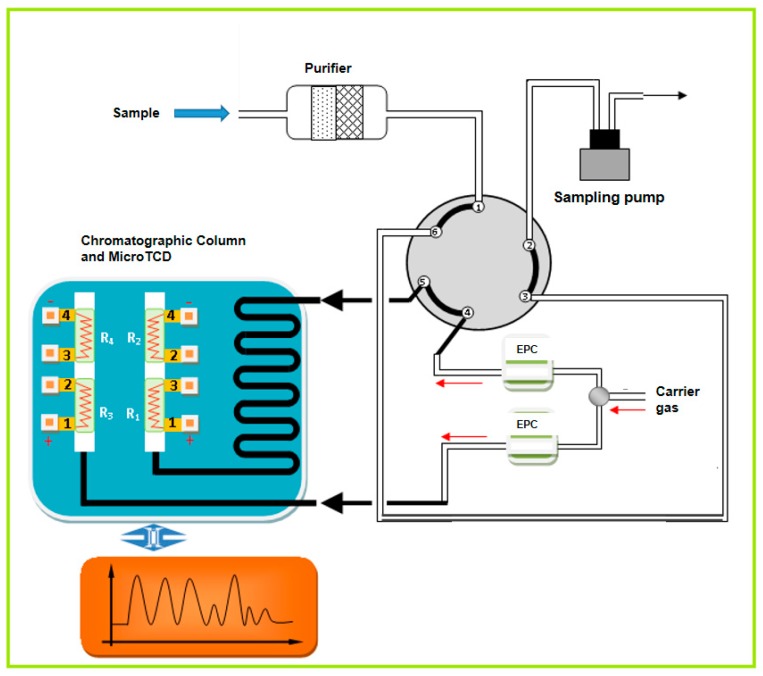
The structural diagram of the GC-μTCD system.

In this work, a portable gas chromatography system integrated with a GC column and μTCD was proposed for monitoring small molecular gas. The proposed system (refer to Figure 8) was mainly composed of sample pretreatment, carrier gas, pneumatic control system, chromatographic column, μTCD, signal acquisition, and a processing module. To obtain high sensitivity and working life, a constant current of 75 mA was applied to the sensor. H_2_ was used as carrier gas due to its high thermal conductivity, and the flow rate of the carrier gas was precisely controlled by the EPC valve. The injection volume was controlled by a 0.2 mL quantitative ring. The dissolved gas was quickly separated by chromatographic column and then transported into the detector by carrier gas, so as to realize accurate quantitative detection of each component.

### 4.2. Detection of Dissolved Gas

**Figure 9:**
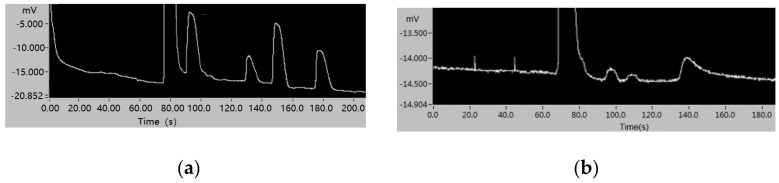
(**a**) The chromatogram of CH_4_, C_2_H_2_, C_2_H_4_, and CO. (**b**) The chromatogram of C_2_H_2_, C_2_H_6_, and CO_2_.

In this work, in order to evaluate that the developed system has the ability to detect trace samples, two standard samples were used for experiments. In the experiment, the samples were dissolved into the naphthenic transformer oil and then extracted and detected. Firstly, a sample containing four kinds of dissolved gases was used to perform the separation and detection performance of the system, where the concentrations of CH_4_, C_2_H_2_, C_2_H_4_, and CO were 109.2 ppm, 95.7 ppm, 115.6 ppm, and 106.1 ppm, respectively. The experiment was performed under isothermal conditions at 80 °C with carrier gas velocity of 5 mL/min. Figure 9a shows the chromatogram of the four dissolved gases, which show that these trace components are highly responsive and effectively detected.

In addition, three low-concentration components (C_2_H_2_, C_2_H_6_, and CO_2_ with concentrations of 9.0 ppm, 9.5 ppm, and 11 ppm, respectively) were used to evaluate the minimum detectable concentration of the system. The experiment was performed under isothermal conditions at 90 °C with carrier gas velocity of 5 mL/min. Figure 9b shows the chromatogram of the system for the detection of these three trace components and the results show that the minimum detected concentration of dissolved gas in oil can be less than 10 ppm.

### 4.3. Quantitative Repeatability of Sensors

In order to evaluate the quantitative repeatability (relative standard deviation, RSD) of the sensor, eight consecutive experiments were carried out under the same experimental conditions with 105.0 ppm ethane at the carrier gas velocity of 5 mL/min. Table 4 indicates the retention time and peak area of chromatographic peaks obtained from these experiments. According to these data, the RSD of retention time was 0.37% and RSD of the peak area was 1.10%, which proved that the developed detector has high stability and consistency.

## 5. Conclusions

In this work, a high sensitivity micro-thermal conductivity detector (μTCD) with four-cell was successfully developed. The microstructure and loading conditions of the detector were successfully optimized. This fabricated μTCD had the advantages of smaller dead volume, high detection sensitivity, and good stability. Compared with Agilent 3000, the developed system reaches the same level in detection limit, analysis time, RSD, and other core indicators. Therefore, the μTCD can be widely used in power transformer fault diagnosis, natural gas and oil exploration, industrial waste gas monitoring, and other fields.

## Figures and Tables

**Table 1:** Thermal stress on the beam varies with thickness.

	1	2	3	4
**Thickness (μm)**	0.4	0.6	0.8	1
**Maximum Stress (MPa)**	33.4	15.2	10.3	7.14

**Table 2:** Maximum stress of thermistors at different gas velocity.

	1	2	3	4	5	6
**Gas Velocity (mL/min)**	0	2	4	6	8	10
**Maximum Stress (MPa)**	0.13	2.5	5.43	8.42	11.3	14.1

**Table 3:** Mechanical properties of the SiN.

Material	Young’s Modulus (GPa)	Residual Stress (MPa)	Bending Strength (GPa)
**SiN**	308.4 ± 24.1	252.9 ± 32.4	6.2 ± 1.3

**Table 4:** The retention time and peak area of chromatographic peaks obtained from 8 repeated experiments.

	1	2	3	4	5	6	7	8
**Retention Time (*t_r_*)**	150.6	151.1	152.1	150.2	151.6	152.0	151.2	151.3
**Peak Area (mV × *t_r_*)**	63.66	64.68	65.54	63.84	65.38	65.80	64.78	64.96
